# Invasive Colon Cancer Cells Induce Transdifferentiation of Endothelium to Cancer-Associated Fibroblasts through Microtubules Enriched in Tubulin-β3

**DOI:** 10.3390/ijms20010053

**Published:** 2018-12-23

**Authors:** Marta Ewelina Wawro, Katarzyna Chojnacka, Katarzyna Wieczorek-Szukała, Katarzyna Sobierajska, Jolanta Niewiarowska

**Affiliations:** 1 Department of Molecular Cell Mechanisms, Medical University of Lodz, Mazowiecka 6/8, 92-215 Lodz, Poland; marta.wawro@umed.lodz.pl (M.E.W.); katarzyna.chojnacka@stud.umed.lodz.pl (K.C.); katarzyna.wieczorek@umed.lodz.pl (K.W.-S.); 2 Department of Biochemistry, Medical University of Lodz, Mazowiecka 6/8, 92-215 Lodz, Poland; 3 Department of Endocrinology and Metabolic Diseases, Medical University of Lodz, Rzgowska 281/289, 93-338 Lodz, Poland

**Keywords:** tubulin-β3, TGF-βs, CAFs

## Abstract

Colon cancer, the second leading cause of cancer-related deaths in the world, is usually diagnosed in invasive stages. The interactions between cancer cells and cells located in their niche remain the crucial mechanism inducing tumor metastasis. The most important among those cells are cancer-associated fibroblasts (CAFs), the heterogeneous group of myofibroblasts transdifferentiated from numerous cells of different origin, including endothelium. The endothelial-to-mesenchymal transition (EndMT) is associated with modulation of cellular morphology, polarization and migration ability as a result of microtubule cytoskeleton reorganization. Here we reveal, for the first time, that invasive colon cancer cells regulate EndMT of endothelium via tubulin-β3 upregulation and its phosphorylation. Thus, we concluded that therapies based on inhibition of tubulin-β3 expression or phosphorylation, or blocking tubulin-β3’s recruitment to the microtubules, together with anti-inflammatory chemotherapeutics, are promising means to treat advanced stages of colon cancer.

## 1. Introduction

Colon cancer, the second leading cause of cancer-related deaths in the world, is usually diagnosed in invasive stages. Therefore, an understanding of processes induced in late stages of cancer progression is critical for effective treatment of patients suffering from metastatic colorectal cancer. The crucial factors promoting tumor metastasis are interactions between cancer cells and cells located in their niche, such as cancer-associated fibroblasts, tumor-associated macrophages, dendritic cells or platelets [1]. The interactions between platelets and tumor favor metastasis. They might interact with each other through cluster of differentiation 97 (CD97) which leads to granule secretion, including the release of ATP, a mediator of endothelial junction disruption. Lysophosphatidic acid (LPA) secreted by platelets induce tumor invasiveness via proximal CD97-LPAR heterodimer signaling, coupling coincident tumor cell migration and vascular permeability to promote transendothelial migration. This process blocks tumor CD97 receptor as an approach to ameliorate metastatic spread [2]. The most important group of the cells which promote metastasis is a heterogeneous group of cancer-associated fibroblasts (CAFs). CAFs derive from cells of different origin, including endothelium, through induction by numerous cytokines and growth factors secreted by cancer or cells located in the cancer niche. They are a key component of tumor stroma inducing cancer growth and invasiveness through the extracellular matrix (ECM) structure modulation, tumor metabolism and immunological reprogramming [3]. The process of endothelial cell transdifferentiation known as an endothelial-to-mesenchymal transition (EndMT) plays a significant role in the cancer progression. It is associated with modulation of cellular morphology, polarization and migration ability as a result of microtubule cytoskeleton reorganization.

Microtubules, highly dynamic structures of the cytoskeleton, contribute to maintaining the structure of the cell and coordinate cell migration. They make up the internal structure of cilia and flagella enabling their movement. Microtubules provide platforms for intracellular transport of proteins, secretory vesicles, organelles, and intracellular macromolecular assemblies (via dynein and kinesin) [4]. Microtubules are composed of dimers of alpha (α) and beta (β) tubulins. β-tubulins are characterized by a high degree of structural homology. The differences between tubulin sequences observed at the carboxyl terminus of the molecule are responsible for their interactions with α-subunits or other microtubule-associated proteins. Those regions contain sites of post-translational modification, ensuring functional specificity of the tubulin isotype. Mutation within β-tubulin sequences or their expression alteration can trigger chemoresistance, both directly by reducing drug affinity to microtubules, and indirectly through changes in their dynamics [5].

The β-tubulin family can undergo a series of post-translational alterations, i.e., glycosylation, tyrosination, acetylation, acylation and phosphorylation [4]. Among all tubulins, particularly important is tubulin-β3, which is abundantly expressed in cancer cells. Recently, tubulin-β3 has been also regarded as a tumor invasiveness marker associated with epithelial-mesenchymal transition (EMT) [6]. Studies on colon adenocarcinoma cell lines induced to EMT have shown that elevated tubulin-β3 level is functionally associated with the increased cell migration and invasiveness. EMT is a metastasis-involved process in which cells lose their epithelial character and become invasive. Additionally, post-transcriptional modifications (phosphorylation, glycosylation) regulate tubulin-β3 cellular localization and its phosphorylation may be involved in the modulation of cell motility [7,8].

In the present study, we investigated the influence of colon cancer cells in the modulation of microtubule cytoskeleton during EndMT of Human Microvascular Endothelial (HMEC-1) cells. We have investigated the role of cytokines released in response to TGF-β in invasive colon cancer cells. Moreover, we have investigated whether microtubule structure dependent on TGF-β might regulate the mesenchymal transdifferentiation.

## 2. Results

### 2.1. The Invasive Colon Cancer Stages Induct Tubulin-β3 Overexpression in EndMT Induced HMEC-1 Cells

**Figure 1:**
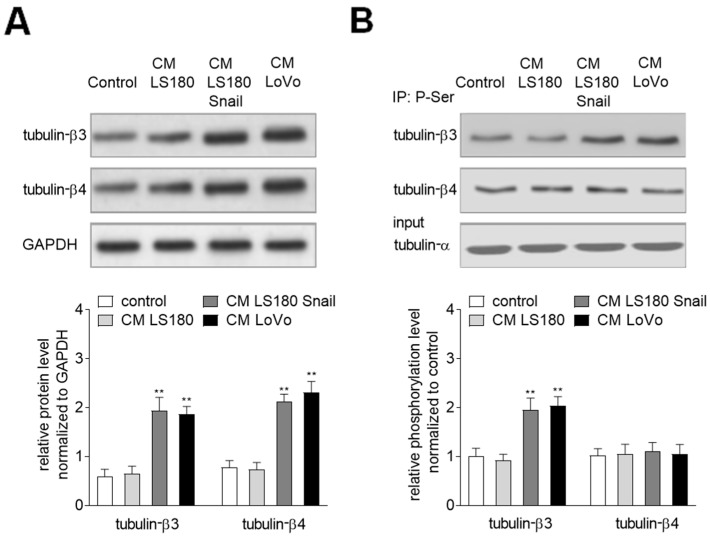
Conditioned media (CM) isolated from invasive (LS180 Snail, LoVo) colon cancer cells induce upregulation of tubulin-β3 and tubulin-β4 in Human Microvascular Endothelial (HMEC-1) cells. (**A**) HMEC-1 cells were cultured in medium supplemented with CM isolated from pre-invasive (LS180) and invasive (LS180 Snail, LoVo) cancer cells for 216 h, and then the levels of tubulin-β3 and tubulin-β4 were analyzed by Western blot assay. The protein levels are normalized to GAPDH. The results are provided as means ± SD (*n* = 3); ** *p* < 0.01. The blots are representative of three independent experiments. (**B**) Simultaneously, the phosphorylation of tubulin-β3 and tubulin-β4 in microtubules fraction was determined by immunoprecipitation assay with rabbit antibodies recognizing phosphorylated proteins followed by Western blot with mouse antibodies bound to tubulin-β3 or -β4. Additionally, the input was analyzed with mouse antibodies recognizing tubulin-α. The results are provided as means ± SD (*n* = 3); ** *p* < 0.01.

Previously, we have shown the ability of invasive colon cancer cells in the induction of EndMT in HMEC-1 cells [8]. Here, we evaluated the level of beta-tubulin subunits in HMEC-1 cells cultured with conditioned media (CM) from invasive (LS180 Snail and LoVo) and pre-invasive colon cancer cells (LS180). The LS180 is the well-described cell line isolated from pre-invasive colon cancer cells (grade II) whereas LoVo is the cell line isolated from a patient who had an invasive form of colon cancer (grade III). LS180 Snail clones were made and well-characterized in our laboratory [8]. We confirmed that Snail overexpression induced characteristic EMT changes like upregulation of mesenchymal markers, downregulation of epithelial markers, cell elongation, higher migration and invasion abilities. All the lines are used in our laboratory as the models of pre-invasive (LS180) and invasive (LS 180 Snail and LoVo) colon cancer cell lines. Based on Western blot assay we demonstrated upregulation of tubulin-β3 (3-fold higher) and tubulin-β4 (2.5-fold higher) in the cells maintained in CM obtained from invasive colon cancer cells (Figure 1A). Additionally, the immunoprecipitation assay showed elevated (about 2-fold higher) phosphorylation of tubulin-β3 in microtubules isolated from cells grown in CM collected from invasive colon cancer cells (Figure 1B). That effect was not observed in HMEC-1 maintained in the medium from pre-invasive colon cancer cells (LS180). In contrast, no changes in phosphorylation of tubulin-β4 were observed in all analyzed cells grown in any CM.

### 2.2. Alteration of Tubulin Level is Dependent on TGF-β Stimulation

**Figure 2:**
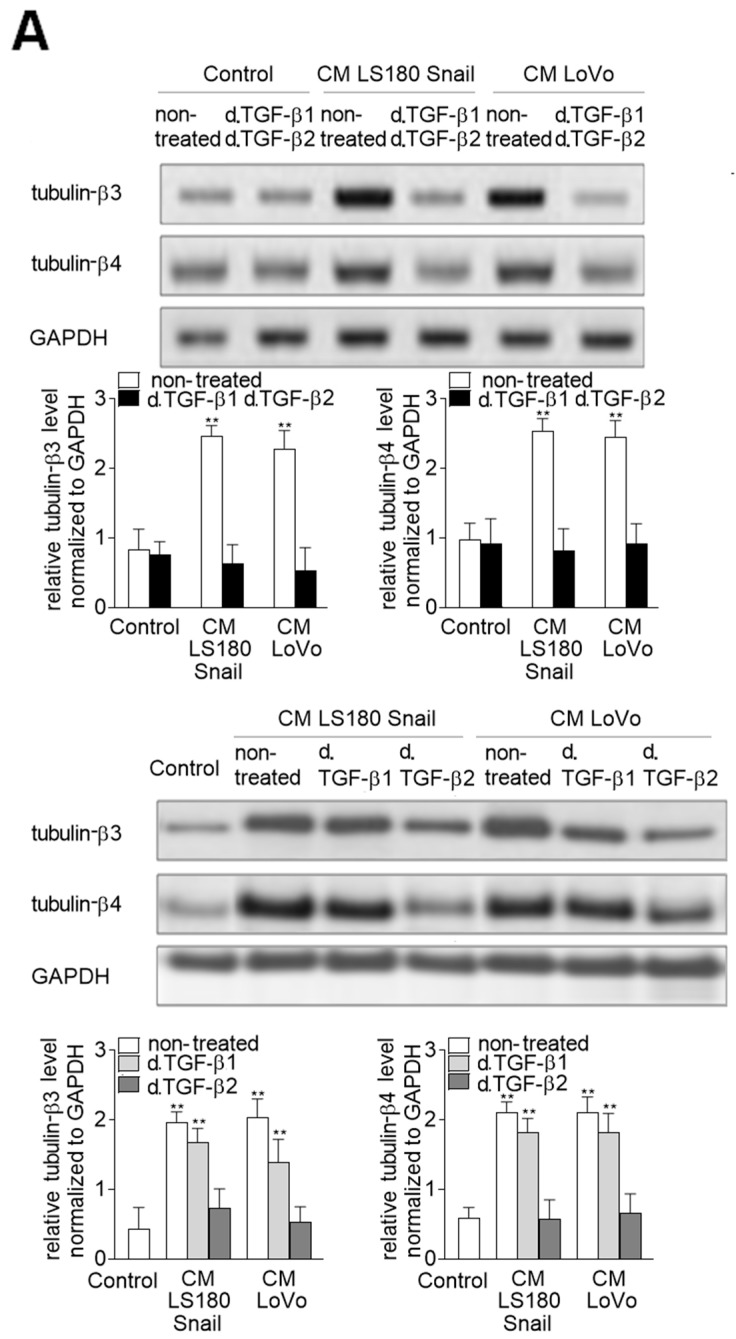

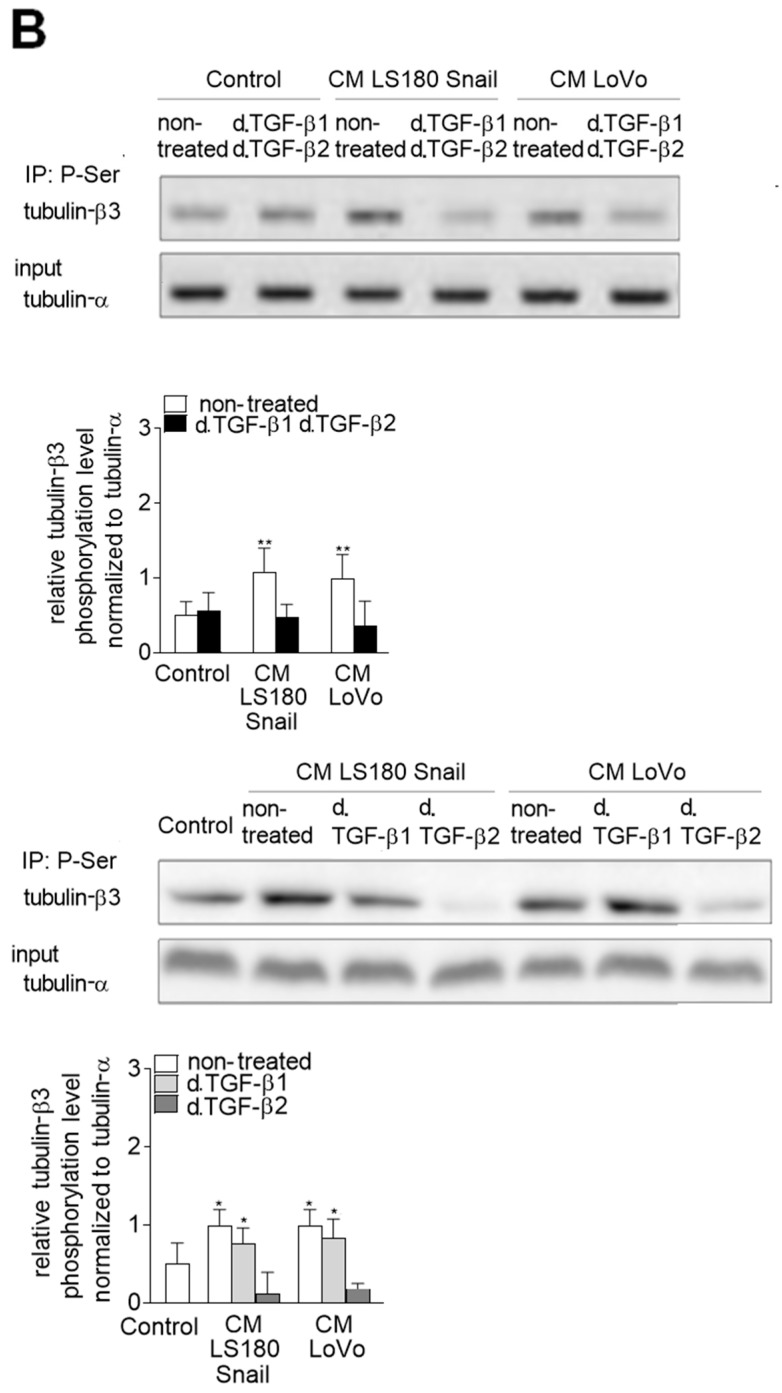
The expression of tubulin-β3 and -β4 are mainly regulated by cytokines belonging to the transforming growth factor-β (TGF-β) family. (**A**) HMEC-1 cells were cultured in medium supplemented with conditioned medium (CM) isolated from invasive (LS180 Snail, LoVo) colon cancer cells where TGF-β1 and/or TGF-β2 were depleted (d. TGF-β1 and/or d. TGF-β2) for 216 h. Then levels of tubulin-β3 and tubulin-β4 were analyzed by Western blot assay. The protein levels are normalized to GAPDH. The results are provided as means ± SD (*n* = 3); ** *p* < 0.01. The blots are representative of three independent experiments. (**B**) Simultaneously, the phosphorylation of tubulin-β3 in microtubules fraction was determined by immunoprecipitation assay with rabbit antibodies recognizing phosphorylated protein followed by Western blot with mouse antibodies bound to tubulin-β3. Additionally, the input was analyzed with mouse antibodies recognizing tubulin-α. The results are provided as means ± SD (*n* = 3); * *p* < 0.05, ** *p* < 0.01.

On the basis of our previous studies [9,10] we decided to check the effect of TGF-β stimulation on tubulin level in EndMT induced cells. This part of our studies was made exclusively with CM obtained from invasive colon cancer cell lines because previous analysis with CM obtained from pre-invasive colon cancer cells did not show any significantly important changes. First, we revealed that depletion of both TGF-β1 and TGF-β2 from conditioned medium abrogated the effect of upregulation of tubulin subunit level in HMEC-1 cells (Figure 2A). The levels of TGF-β1 and TGF-β2 in CM from LS180, LS180 Snail and LoVo, as well as the effectiveness of particular TGF-β depletion, are shown in the supplementary data (Figure S1). Detailed studies showed that reduction of TGF-β2 in CM abrogated tubulin-β3 and -β4 level upregulation was stronger than after TGF-β1 depletion (Figure 2A). Next, we showed that depletion of both TGF-β or only TGF-β2 caused decreased (about 0.65-fold decrease) phosphorylation level of tubulin-β3 in microtubules in comparison to the cells maintained in CM (Figure 2B). Simultaneous studies revealed that reduction of TGF-β1 does not affect the tubulin-β3 phosphorylation in microtubules (Figure 2B).

### 2.3. Phosphorylation of Tubulin-β3 Induce Enhanced Mesenchymal Behavior

**Figure 3:**
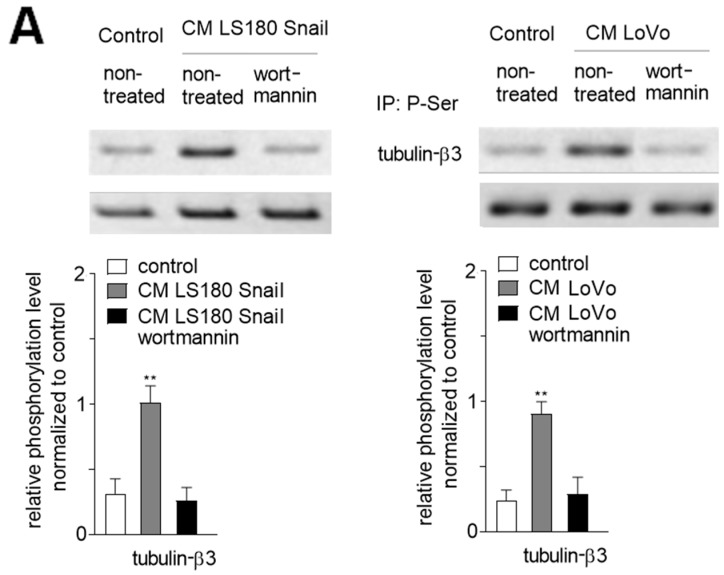

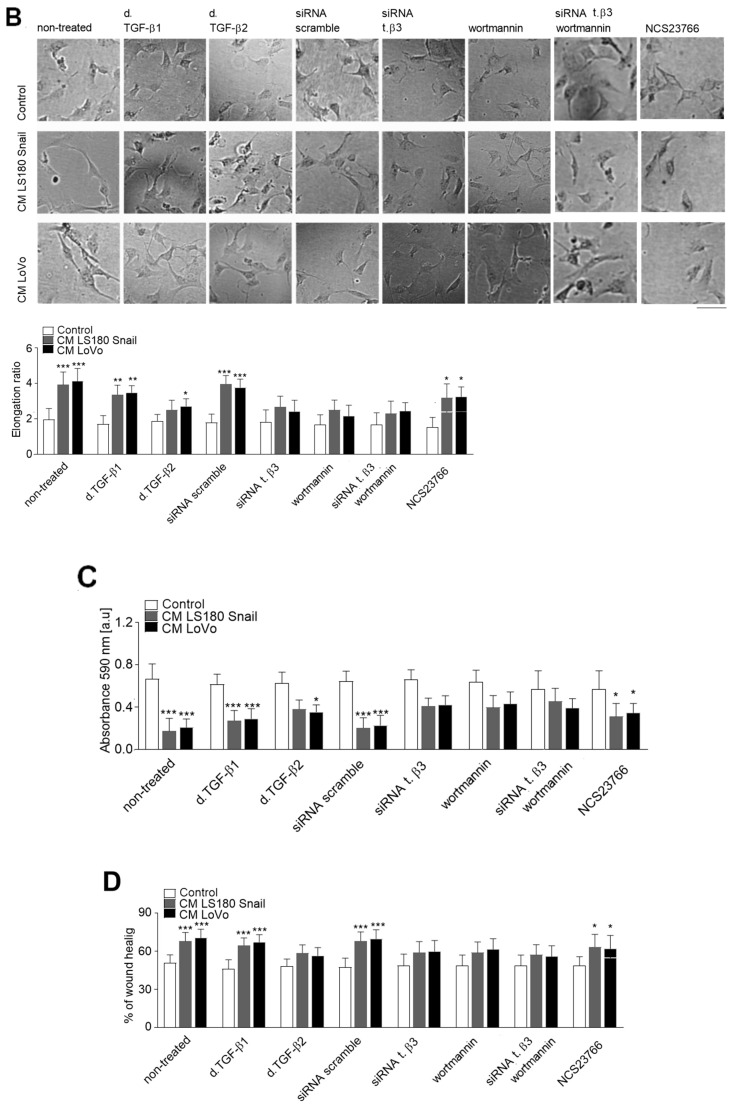
Phosphorylation of microtubular tubulin-β3 regulates CAF behavior. HMEC-1 cells were cultured in medium supplemented with conditioned medium (CM) isolated from invasive (LS180 Snail, LoVo) colon cancer cells where TGF-β1 or TGF-β2 were depleted (d. TGF-β1 or d. TGF-β2) for 216 h or where tubulin-β3 was silenced (siRNA t. β3), or phosphorylation of tubulin-β3 was inhibited by wortmannin. Additionally, in tubulin-β3-silenced cells (siRNA t. β3) the phosphorylation of tubulin-β3 was inhibited by wortmannin or Rac1 was inhibited by NCS23766. Then, the phosphorylation of tubulin-β3 in microtubule fraction from wortmannin treated-cells maintained in CM from invasive colon cancer cells (LoVo and LS180 Snail) (**A**) was determined by immunoprecipitation assay with rabbit antibodies recognizing phosphorylated protein. Next, the Western blot with mouse antibodies binding to tubulin-β3 was performed. Additionally, the input was analyzed with mouse antibodies recognizing tubulin-α. The morphology (20×) (**B**), adhesion to collagen I (**C**) and wound-healing properties (**D**) were analyzed. Additionally, the elongation ratio in 50 randomly chosen cells was measured and is shown. The results are provided as means ± SD (*n* = 3); * *p* < 0.05, ** *p* < 0.01, *** *p* < 0.005. t. β3—tubulin-β3.

The analysis of cell behavior showed that blocking tubulin-β3 phosphorylation via TGF-β2 depletion in medium supplemented with conditioned medium (CM) isolated from invasive (LS180 Snail, LoVo) colon cancer cells caused partial inhibition of cell elongation, as well as slower cell migration in comparison to cells maintained in CM obtained from invasive colon cancer cells. Detailed studies showed that cells grown in the medium from invasive cells were almost two-times longer than control cells. The reduction of TGF-β1 level in CM from invasive cells resulted in slightly marked inhibition of cell elongation, while TGF-β2 depletion led to the more than half-lower ability of cell elongation in comparison to CM-induced cells. A similar effect was observed after tubulin-β3 expression silencing or inhibition of its phosphorylation by wortmannin treatment (Figure 3B). Simultaneously, immunoprecipitation analysis of wortmannin-treated cells grown in CM medium from invasive colon cancer cells showed about a 75% decrease of phosphorylated tubulin-β3 (Figure 3A). To determine the optimal siRNA concentration efficiency in silencing tubulin-β3 expression, we tested different concentrations (25, 50, 75, 100 nM) of an antisense oligonucleotides specific to tubulin-β3 using real-time PCR. On the basis of the obtained results, a concentration of 75 nM was chosen (Figure S2A). Next, the effect of tubulin-β3-silencing by 75 nM siRNA was tested in each experiment (Figure S2B). Cells cultured in CM from invasive colon cancer cells demonstrated about 2.5-fold lower adhesive properties to collagen I in comparison to control endothelial cells (Figure 3C). Depletion of TGF-β2 from CM caused in half abrogation that effect. Silencing of tubulin-β3 expression or wortmannin-treatment partially restored adhesion capacity. Finally, microscopic analysis of wound-healing properties (Figure 3D and Figure S3) demonstrated that CM-dependent stimulation resulted in about 24% faster migration. Tubulin-β3 expression silencing, wortmannin-treatment or TGF-β2 abrogation from CM resulted in half-lifting that effect, whereas depletion of TGF-β1 caused 6% inhibition of cell migration on collagen I in comparison to the effect observed within the cells grown in CM (Figure 3D and Figure S3). Additionally, to confirm that inhibition of phosphatidylinositol-3-kinase (PIK3) regulates EndMT through tubulin-β3 phosphorylation we performed an analysis where expression of tubulin-β3 was silenced and, simultaneously, the phosphorylation of tubulin-β3 by PI3K was inhibited by wortmannin treatment. A similar analysis with Rac1 inhibitor NCS23766 was performed to check the role of tubulin-β3 phosphorylation in EndMT. A comparison of these studies showed that inhibition of Rac1 activity by NCS23766 caused fewer visible differences in cell morphology, as well as cell behavioral modulation analysis. The silencing of tubulin-β3 resulted in modulation similar to that observed after wortmannin treatment in all presented assays (Figure 3B–D and Figure S3).

### 2.4. Microtubules Enriched in Phosphorylated Tubulin-β3 are Critical for Alteration of Contraction Protein Expression

**Figure 4:**
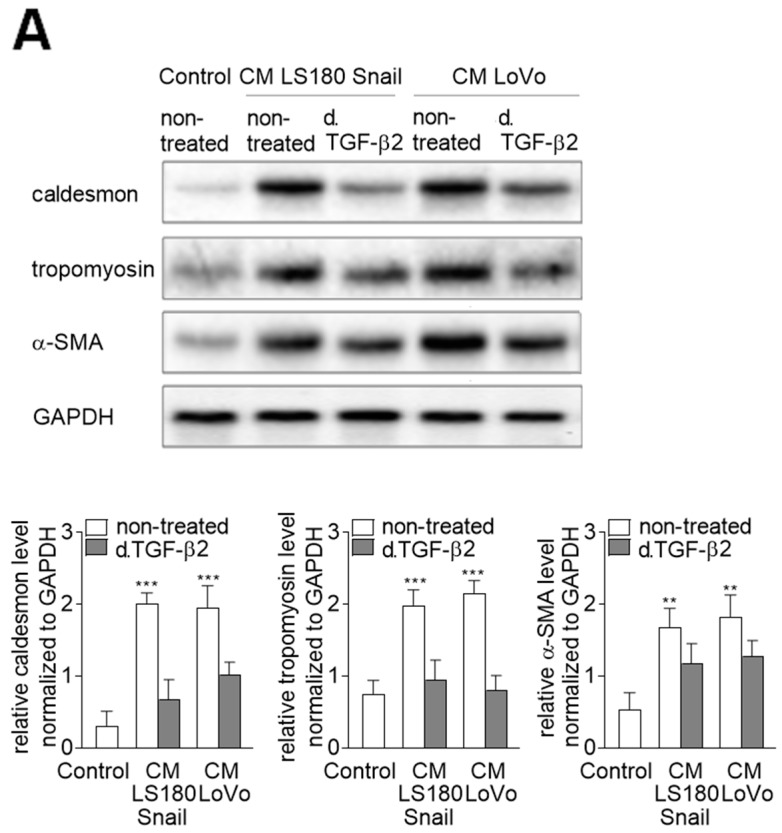

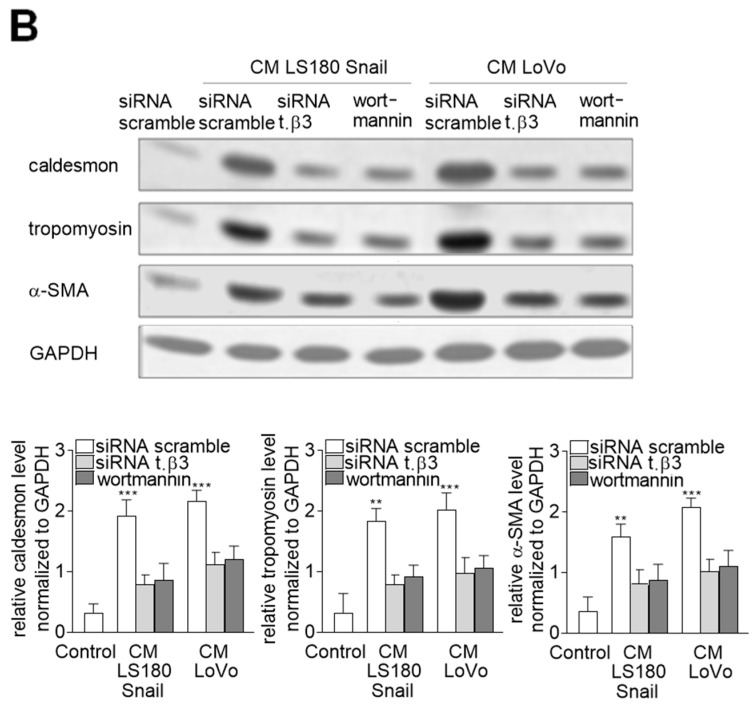
Phosphorylation of tubulin-β3 in CAF-like cells is the response for contraction protein expressions. (**A**) HMEC-1 cells were cultured in medium supplemented with conditioned medium (CM) isolated from invasive (LS180 Snail, LoVo) colon cancer cells where TGF-β2 was depleted (d. TGF-β2) for 216 h or where (**B**) tubulin-β3 were silenced (siRNA t. β3), or phosphorylation of tubulin-β3 were inhibited by wortmannin. Then, the levels of contraction proteins (caldesmon, tropomyosin, and α-SMA) were determined by Western blot assay. The protein levels are normalized to GAPDH. The results are provided as means ± SD (*n* = 3); ** *p* < 0.01, *** *p* < 0.005. The blots are representative of three independent experiments.

In further experiments, we studied the effect of tubulin-β3 presence on the regulation of contraction protein expression in EndMT-induced HMEC-1 cells. That part of our studies (Figure 4A) reveals exclusively effects of TGF-β2 depletion because TGF-β1-treatment did not present any significantly important changes. Previously, we showed that α-SMA, caldesmon, and tropomyosin were strongly upregulated during EndMT [9]. Here we depleted TGF-β2 from CM isolated from LS180 Snail cells (Figure 4A) and observed about 2-fold lower level of caldesmon and tropomyosin. The effect on α-SMA expression was less visible and lead to the 0.5-fold decrease of protein level in comparison to CM stimulated cells (Figure 4A). These modulations were similar to those observed in the cells stimulated by CM from invasive colon cancer cell line LoVo (Figure 4A). To confirm that those processes might be regulated by tubulin-β3, we silenced its expression by siRNA strategy or inhibited its phosphorylation by wortmannin treatment (Figure 4B). We showed that downregulation of tubulin-β3 or its phosphorylation caused a similar effect to that observed after TGF-β2 depletion.

### 2.5. Microtubules Enriched in Phosphorylated Tubulin-β3 Regulate Rac1 Activation

**Figure 5:**
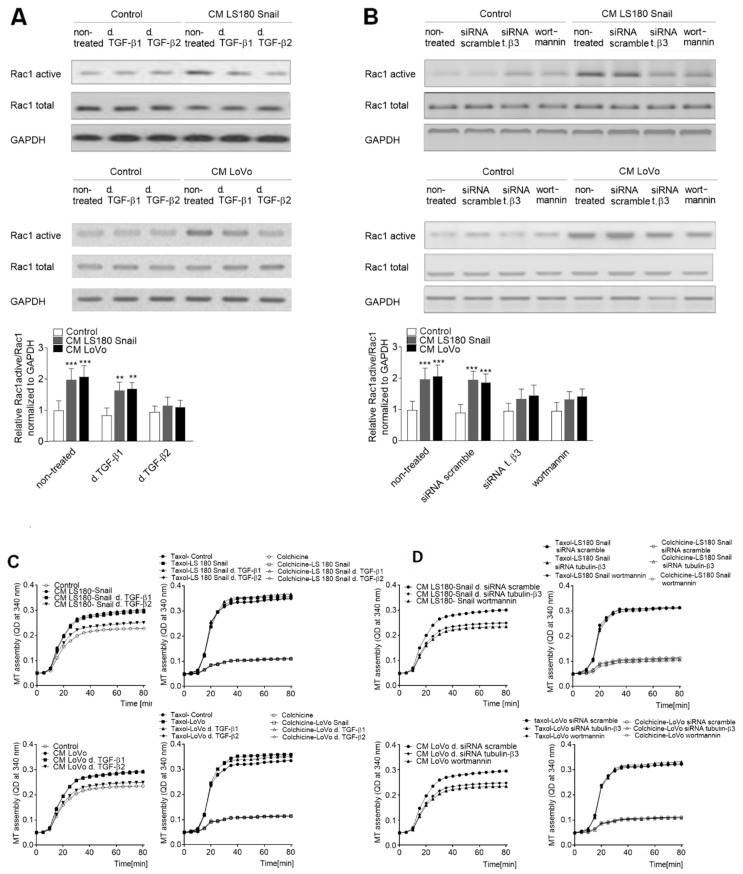

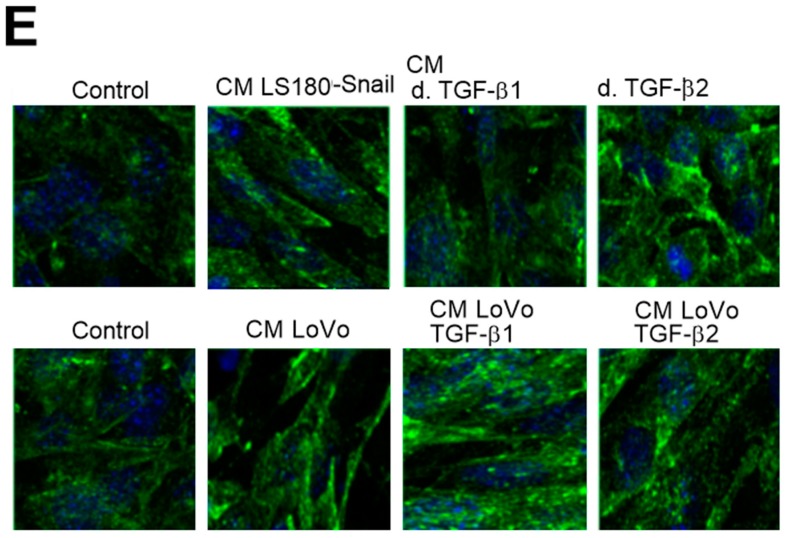
Phosphorylation of tubulin-β3 in microtubules of CAF-like cells is engaged in EndMT via regulation of Rac1 activity. HMEC-1 cells were cultured in medium supplemented with conditioned medium (CM) isolated from invasive (LS180 Snail, LoVo) colon cancer cells where TGF-β1 or TGF-β2 was depleted (d. TGF-β1 or d. TGF-β2) for 216 h (**A**) or tubulin-β3 were silenced (siRNA t. β3) or phosphorylation of tubulin-β3 was inhibited by wortmannin (**B**). Then, the level of Rac1 active form was determined by Western blot assay. The protein levels are normalized to GAPDH. The results are provided as means ± SD (*n* = 3); ** *p* < 0.01, *** *p* < 0.005. The blots are representative of three independent experiments. t. β3 - tubulin-β3. Additionally, microtubule polymerization was analyzed in HMEC-1 cells cultured in medium supplemented with CM isolated from invasive (LS180 Snail, LoVo) cancer cells where TGF-β1 or TGF-β2 was depleted (d. TGF-β1 or d. TGF-β2) for 216 h (**C**) or tubulin-β3 was silenced or phosphorylation of tubulin-β3 was inhibited by wortmannin (**D**). Controls of microtubules polymerization with taxol and depolymerization with colchicine were conducted and are shown in the right graphs (**C**, **D**). Next, confocal microscopy (63×) of microtubules was conducted with antibodies specifically bound to tubulin-α-FITC (green) and nucleus were labelled by 4′,6-diamidino-2-phenylindole (DAPI) (blue) (**E**).

Next, we sought to discover the pathway regulated by tubulin-β3 expression or microtubules polymerization involved in EndMT induction. It has been known that Rac1 activation observed during EndMT controls microtubules polymerization in fibroblasts (regulation of leading-edge microtubule and actin dynamics downstream of Rac1 [11]). Rac1 is also activated during mesenchymal transdifferentiation, thus we decided to analyze that process. First, we analyzed the level of activated Rac1 in cells maintained in CM and showed a decrease of Rac1 activation where TGF-β1 (0.2-fold decrease) or TGF-β2 (0.5-fold decrease) were depleted (Figure 5A). Then, to confirm our hypothesis we inhibited phosphorylation of tubulin-β3 by wortmannin-treatment as well as its expression by silencing assay. We revealed that in both analyzed cases activation of Rac1 was significantly lower (0.4-fold decrease) (Figure 5B). Additionally, we checked the ability to polymerize in those samples by tubulin polymerization assays in vitro. We demonstrated that microtubules isolated from cells grown in CM from invasive colon cancer cells (LS-Snail and LoVo) manifested faster polymerization ability. Depletion of TGF-β2 from CM resulted in a decrease of polymerization rate of microtubules and was similar to that observed in control non-treated endothelial cells. In contrast, depletion of TGF-β1 from CM did not induce such changes (Figure 5C). Additionally, confocal microscopy analysis showed that depletion of TGF-β2 level particularly resulted in microtubule structure changes in vivo and, finally, in modulation of cell morphology to a more rounded one (Figure 5E). Polymerization of microtubules isolated from cells growing in CM from invasive colon cancer cells where tubulin-β3 was silenced or its phosphorylation was inhibited by wortmannin also revealed a decrease of polymerization ability. It was especially observed in wortmannin-treated cells (Figure 5D).

**Figure 6:**
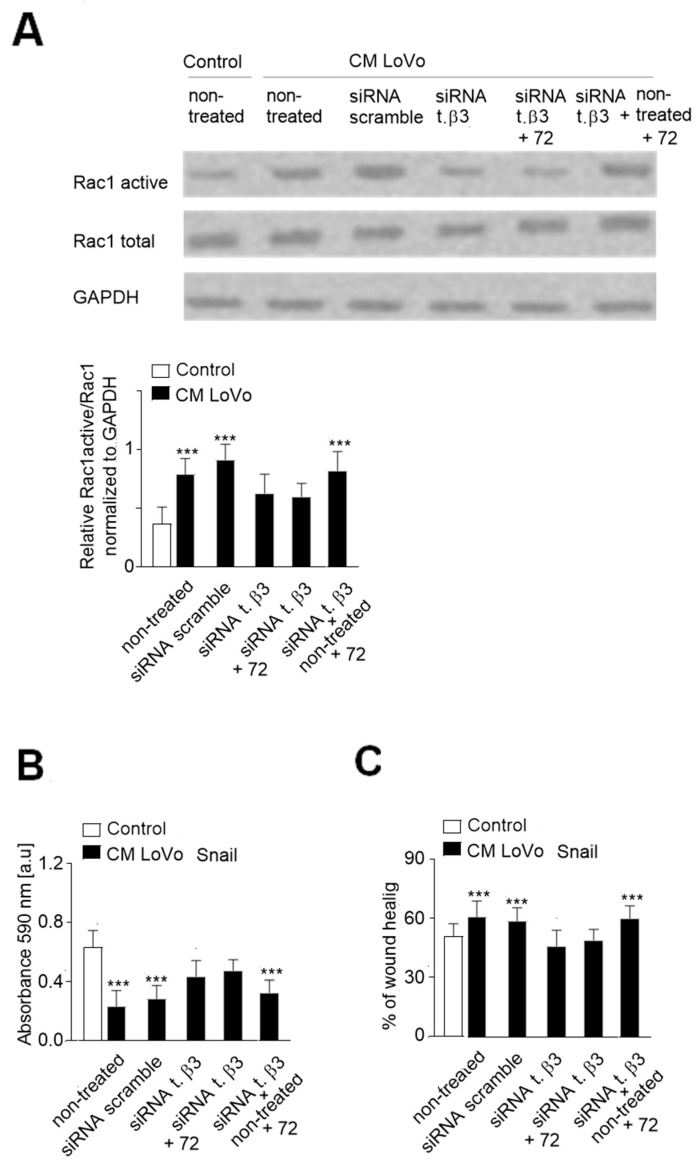
Microtubular tubulin-β3 regulates CAF behavior via Rac1. HMEC-1 cells were cultured in medium supplemented with conditioned medium (CM) isolated from invasive (LoVo) colon cancer cells where tubulin-β3 was silenced (siRNA t. β3). Then, silencing was halted to check the effect of tubulin-β3 on Rac1 activation and cell behavior. The Rac1 activity was analyzed in control, CM-treated and tubulin-β3 silenced cells and in the recovery experiments where silencing was halted for 72 h (**A**). GAPDH was used as a loading control. The results are shown as means ± SD (*n* = 3); *** *p* < 0.005. Next, the adhesion to collagen I (**B**) and wound-healing properties (**C**) were analyzed. The results are shown as means ± SD (*n* = 3); *** *p* < 0.005. t. β3—tubulin-β3.; +72—the time of the elongated cell treatment.

To confirm our hypothesis that microtubules enriched in phosphorylated tubulin-β3 are involved in EndMT regulation, we analyzed the effect of tubulin-β3 in a recovery assay. Therefore, cells were treated for 72 h longer than in the previous experiment with (as a control of influence) and without siRNA for tubulin-β3. Then the level of Rac1 activity correlating with EndMT process was analyzed. We observed that increased time of tubulin-β3 silencing did not induce a visibly stronger inhibition of Rac1 activity (Figure 6A). In contrast, recovery of tubulin-β3 expression up to the control level (Figure S4) resulted in the increase of Rac1 activity to a level similar to that observed in cells maintained in CM from LoVo cells. We detected that this process correlated with lower adhesion ability (Figure 6B) and increased migration (wound healing) (Figure 6C and Figure S4) properties of HMEC-1 cells compared to tubulin-β3-silenced cells.

## 3. Discussion

The vascular endothelial cells create a tumor niche, manifest considerable plasticity and may transdifferentiate into other cell types under physiological and pathological states such as cancer, fibrosis or arteriosclerosis [12]. Endothelium through the EndMT remains the main source of CAFs [13]. Those perpetually activated fibroblasts are primarily responsible for the development of cancer metastasis [14,15]. Hence, recognizing the role of particular factors, including those secreted by tumor cells, and understanding the pathways responsible for endothelial transdifferentiation in the promotion of CAFs behavior, represents a very attractive target in the search for new effective anti-cancer therapies.

Our studies demonstrated that colon cancer cells influence the regulation of EndMT. That correlation was detected only when endothelial cells were grown in the CM obtained from invasive colon cancer. Phenotypic heterogeneity of endothelial cells, including mesenchymal transdifferentiation, are induced by cytokines and growth factors secreted by tumor cells [16]. EndMT might be induced by numerous factors such as IL 1-β, TNF-α, TGF-β family and NF-κB transcription factor [17,18]. It has been previously shown that the superfamily of transforming growth factor-β (TGF-β) plays a pivotal role in the EndMT in fibrosis development [19] but knowledge about its role in cancer progression is still not satisfactory. Although TGF-β1, as well as TGF-β2, might induce mesenchymal transdifferentiation of HMEC-1 cells, both the elongation of the cell shape and changes in the ability to adhere and migrate to collagen are more associated with TGF-β2 stimulation.

It has been known that the dynamics of the microtubule structures enable alteration of cell adhesion and migration [20,21,22]. The composition of microtubules subunits, especially β-tubulins, is engaged in the stabilization of the filament structures [23]. It has been revealed that microtubules enriched with tubulin-β2 or -β4 are more stable than filaments that possess numerous β3 subunits [24]. The presence of tubulin-β3 is associated with faster polymerization and more frequent microtubular catastrophe [25]. The specificity and necessity of individual β-tubulin isoforms in the regulation of microtubules dynamics, especially tubulin-β3, has been shown for a variety of processes associated with the modulation of cell movements [26,27,28]. Changes of those cellular abilities are observed also in mesenchymal transdifferentiation [25]. Our detailed analysis revealed that CM from both models of invasive colon cancer cell lines (LS180 Snail and LoVo) upregulated two beta-tubulins, tubulin-β3, and -β4, mainly by the cytokines from the TGF-β family. Those results confirming our previous observation where we have shown the cumulation of β3 and β4 within the microtubules, indicates their role in the microtubule rearrangement during EndMT [10]. In contrast, other beta-tubulins did not demonstrate that ability [10]. Additionally, we observed increasing tubulin-β3 phosphorylation during EndMT. Posttranslational modification within beta subunits is involved in the alteration of cell adhesion and migration. Those processes have been observed both in Chinese hamster ovary (CHO) cells and cancer cells [8,29]. Inhibition of PI3K by wortmannin might block other processes and proteins engaged in the EndMT process. One of those is the Rac1 protein or Polo-like-kinases (Plks) [30]. Our analysis, that despite the effect of wortmannin on Rac1 activity the total effect of inhibition of Rac1 and the level of phosphorylated tubulin-β3 is significant. This indicates the participation of phosphorylated tubulin-β3 in the regulation of EndMT. Additionally, Plks, whose activity might be inhibited by wortmannin, are implicated in the microtubule nucleation. Plk1 depletion or inhibition of its phosphorylation prevented the recruitment of γ-tubulin to centrosome [30]. The role of Plk1 was demonstrated in the EMT as the activator of the ERK/MAPK pathway, which is crucial for increasing migration ability of cells that acquire mesenchymal character. [31]. However, its function and expression were not detected in EndMT and in fact, may be interesting to investigate in the future.

Here we noted for the first time that the presence of tubulin-β3 correlated with the higher level of contraction proteins. It has been known that microtubule rearrangement is involved in the regulation of cell morphology. Additionally, microtubules might interact with actin cytoskeleton thus regulating reorganization of cell structures. We previously demonstrated that the ILK-MMP9-MRTF-A pathway regulated that process during EndMT in HMEC-1 cells [9]. The integrin-linked kinase (ILK) function might be also regulated by interaction with microtubules and is dependent on their dynamics [32,33]. We suppose that changes in microtubule composition observed during endothelial cell transdifferentiation have to influence actin cytoskeleton and expression of contraction proteins. That hypothesis requires further extensive studies.

In opposite to abundantly expressed tubulin-β1 or -β2, the presence of tubulin-β3 subunit in physiological conditions is limited to neuronal tissue and testis [34]. It is also detected through the development of solid tumors, such as in breast, ovarian or testis cancer [35]. Initially, it was suggested that the appearance of tubulin-β3 is associated with the acquisition of chemoresistance by tumor cells. The subunits have been known as a marker of taxane- or vinca-alkaloid-based drug resistance in ovary [36], lung [37], stomach [38], pancreas [39] and breast [40] cancer. Tubulin-β3 is not able to be recognize by chemotherapeutics, so its presence prevents interaction between drug and microtubules. Thus, drugs are not able to block karyokinetic spindle and cell division. Based on our previous [10] and current studies, we suggest that higher expression of Tubulin-β3 in CAFs call into question the sense of using taxane-based therapies in advanced stages of colorectal cancer. Chemotherapeutics that do not inhibit cell division and consequently do not block the division of CAFs might actually promote metastasis and spread of cancer.

Recently, tubulin-β3 has also been regarded as a tumor invasiveness marker associated with epithelial-mesenchymal transition (EMT) [4]. On the basis of our previous studies [10] and present observations which indicate an increase of tubulin-β3 expression also during EndMT, we suggest that tubulin-β3 could be perceived more thoroughly, as a biomarker of mesenchymal transdifferentiation. That observation may contribute to the design of new therapeutic approaches for attenuating fibrosis in cardiovascular diseases such as atherosclerosis, pulmonary hypertension, and cardiac disorders. Our findings can be useful in the development of new therapies based on the inhibition of tubulin-β3 expression or phosphorylation. We are convinced that for patients with diseases stemming from mesenchymal cell transdifferentiation, like tumors, fibrotic diseases or cardiomyopathies, such treatment would be of considerable importance.

## 4. Materials and Methods

### 4.1. Cell Cultures

Human microvascular endothelial cells (HMEC-1) were a gift from Prof. Kathryn Keller (Centers for Disease Control and Prevention, Atlanta, GA, USA). The cells were cultured in MCDB131 (Life Technologies, Paisley, UK) medium supplemented with 10% heat-inactivated fetal bovine serum (FBS) (Life Technologies) or with the conditioned medium (CM) from pre-invasive (LS180) or invasive (LoVo, LS180 Snail clones 2 (cl2) or 8 (cl8)) colon cancer cell lines in a humidified 5% CO_2_ atmosphere at 37 °C. CM was recovered from colon cancer cells growing for 72 h. CM was collected, centrifuged to eliminate cells and mixed with MCDB131 medium in a proportion of 1:2. The HMEC-1 cells were cultured with this medium supplemented with CM for 216 h, and the medium was changed every 72 h. In some experiments, cells were treated with wortmannin (1.0 µg/mL, Bio-Rad, Munich, Germany), to inhibit tubulin-β3 phosphorylation or NCS23766, inhibitor of Rac1 (100 nM, Sigma-Aldrich, Steinheim, Germany) activity. Cells were harvested by 0.05% trypsin-EDTA and washed with phosphate-buffered saline (PBS).

### 4.2. Depletion of TGF-β

CM was incubated with IgG binding to TGF-β1 or/and TGF-β2 for 2 h. To confirm TGF-β depletion the level of cytokines was analyzed using sandwich enzyme-linked immunosorbent assay (ELISA) kits (Human TGF-β1 and Human TGF-β2 Quantikine ELISA Kits, R&D Systems, Minneapolis, MN, USA) according to the manufacturer's instructions. The optical density of each reaction was measured at 450 nm using a microplate reader (Bio-Rad) and corrected against absorption at 570 nm.

### 4.3. siRNA Strategy

siRNAs targeting human tubulin-β3 or a negative control siRNA (Dharmacon) were transfected into cells using X-fect^®^ (Clontech, Mountain View, CA, USA) reagent according to the manufacturer's protocol for 33 h.

### 4.4. Cell Morphology

Cell morphology of maintained endothelial cells was analyzed by fluorescence microscopy (Olympus, San Jose, CA, USA). The representative images were captured using an Olympus digital camera and the alterations in cell morphology were estimated by measuring the elongation ratio (the ratio of the longer to the shorter axis) using ImageJ software (NIH, Bethesda, MD, USA).

### 4.5. Adhesion Assay

Wells of F8 Maxisorp loose Nunc-Immuno^TM^ modules (Nunc^TM^ brand products) were coated with 10 μg/mL of collagen type I in Tris-buffered saline (TBS) (0.02 mM Tris/HCl, 0.15 mM NaCl, pH 7.5) for 2 h at 37 °C before the wells were rinsed twice with TBS. Next, the wells were blocked with 1% heat-denatured bovine serum albumin(BSA) in washing buffer (0.1 mM CaCl_2_ in TBS, pH 7.5) for 1.5 h at 37 °C in a humidified 5% CO_2_ atmosphere. The 1.5 × 10^5^ cells were then transferred into plates for 1 h to adhere and non-adherent cells were removed with washing buffer. Next, the cells were fixed with 10% methanol and 10% acetic acid in water for 15 min, stained with 0.5% crystal violet for 10 min, rinsed a few times and dried. Intracellular crystal violet was dissolved by incubating with 10% acetic acid for 10 min. The total amount of cell-associated protein was determined by a microplate reader at 595 nm (Perkin Elmer Wallac 1420 Victor2 Microplate Reader, Waltham, MA, USA).

### 4.6. Wound Healing

Cells were cultured on collagen type I pre-coated on a 12-well plate until reaching 80–90% confluency. When the monolayer was confluent, cells were starved in FBS-free medium for 4 h. Images were then captured immediately after wounding (time 0) and then 8 h later. The migration of cells into the denuded area was visualized using an inverted Nikon phase-contrast microscope (400× magnification) and a digital camera (Olympus IX81). Cell migration was quantified by image analysis of a minimum of 4 randomly-selected fields of view of the denuded area. The mean wound area was expressed as a percentage of recovery (%*R*) from three identically-treated plates using the equation: %*R* = [1 − (*T_t_*/*T_0_*)] × 100, where *T_0_* is the wounded area at 0 h and *T_t_* is the wounded area 8 h post-injury.

### 4.7. Immunoprecipitation of Microtubules

Microtubule proteins were isolated from the cell pellet homogenized in PB buffer (0.1 M K-PIPES (pH 6.8), 0.5 mM MgCl_2_, 2 mM EGTA, 0.1 mM EDTA, 0.1% (*v*/*v*) β-mercaptoethanol, 1 mM ATP with PhosStop phosphatase inhibitor and cOmplete Protease Inhibitor Cocktail) before centrifuging (100,000× *g*, 60 min, 4 °C). Next, the cytosolic supernatants were collected, mixed with a half volume of 100% glycerol preheated at 37 °C with ATP and MgCl_2_ (concentrations of 3.5 mM), polymerized 60 min at 37 °C. Pellets were collected, centrifuged (100,000 × *g*, 45 min, 37 °C), resuspended with ice-cold PB buffer and depolymerized on ice for 30 min. This process was repeated twice. Next, cell proteins were extracted (15 min at room temperature (RT)) with 0.5% NP-40 in a microtubule stabilization buffer containing 20 mM Tris, pH 6.9, 0.5% (*v*/*v*) NP-40, 2 mM glycerol, 10% (*v*/*v*) DMSO, 1 mM MgCl_2_, 2 mM EGTA, 200 mM sodium orthovanadate, 1 mM Phenylmethylsulfonyl fluoride (PMSF) and PhosStop phosphatase inhibitor and cOmplete Protease Inhibitor Cocktail. The detergent-insoluble material was pelleted by centrifugation (15,000 × *g*, 10 min, RT) and soluble extracts were used for immunoprecipitation with appropriate antibodies.

### 4.8. Western Blot Assay

Briefly, exponentially growing cells were lysed in M-PER (Mammalian Protein Extraction Reagent (Thermo Scientific Pierce, Rockford, IL, USA) supplemented with Halt Protease Inhibitor Cocktail (Thermo Scientific Pierce, Rockford, IL, USA) according to the manufacturer's instructions. The whole-cell extracts were collected, aliquoted, and stored at −80 °C until protein quantification with BCA Protein Assay Kit according to the manufacturer's protocol. Proteins from lysates or immunoprecipitates (30 μg) were separated by 10% sodium dodecyl sulfate-polyacrylamide gel electrophoresis (SDS-PAGE; Bio-Rad), electroblotted (120 min, 200 mA, 4 °C) onto nitrocellulose membranes (Bio-Rad) and blocked for 2 h with Tris-buffered saline (TBS) containing 5% BSA at room temperature. Next, the blot was incubated with primary antibodies overnight at 4 °C, washed and incubated with anti-rabbit or anti-mouse horseradish peroxidase-conjugated secondary antibodies for 1 h at room temperature. Finally, chemiluminescence was detected using Pierce ECL Western Blotting Substrate and Kodak BioMax Light Film. The films were scanned (HP Scanjet G4050 scanner, Palo Alto, CA, USA), and the relative protein levels were quantified by ImageJ software. The background was subtracted, and the area for each protein peak was determined. Protein levels were normalized using an appropriate loading control (GAPDH or tubulin-α).

### 4.9. Confocal Microscopy

1 × 10^5^ cells were placed on sterile glass microscope slides and cultured at 37 °C in a humidified atmosphere of 5% CO_2_. 48 h later, the cells were washed with phosphate-buffered saline (PBS), fixed with 4% formaldehyde in PHEM buffer (60 mM Pipes, pH 6.9, containing 25 mM Hepes, 10 mM EGTA, 4 mM MgCl_2_ and cOmplete™, Mini, EDTA-free Protease Inhibitor Cocktail) for 20 min at room temperature and prepared as described previously [9]. The cells were then washed 3 times with PHEM buffer, permeabilized with 0.1% Triton X-100 (*v*/*v*) and blocked with 2% (*v*/*v*) BSA in PHEM buffer at room temperature for 60 min. The tubulin-α localization were detected by cell staining with antibodies (1:200) conjugated with Fluorescein isothiocyanate (FITC) at 37 °C for 60 min. Nucleus in each cell was labeled by DAPI during 30 min. A Leica TCS SP8 confocal laser microscope system was used for intracellular probe visualization. Series of single 0.2 μm optical sections were collected. The images were scanned at high resolution (63× oil objective, 1.4 numerical aperture (NA)).

### 4.10. Statistical Analysis

The results are presented as the mean of at least 3 independent experiments ± standard error. Statistical significance of the differences between the experimental conditions was determined by one-way ANOVA followed by Tukey’s test (GraphPad Prism Software, 8.0.0 for Windows, San Diego, CA, USA). Differences between means were considered significant when *p*  ≤  0.05.

## Supplementary Materials

Supplementary materials can be found at http://www.mdpi.com/1422-0067/20/1/53/s1.

## Abbreviations

CAFs	Cancer-Associated Fibroblasts
EndMT	Endothelial-Mesenchymal Transition
HMEC-1	Human Microvascular Endothelial Cells
TGF-β	Tumor Growth Factor-β
CM	Conditioned Medium
t.β3	Tubulin-β3
ECM	Extracellular Matrix
