# Supplementary material for: Invasive Colon Cancer Cells Induce Transdifferentiation of Endothelium to Cancer-Associated Fibroblasts through Microtubules Enriched in Tubulin-β3
Source: Int J Mol Sci. 2018 Dec 23;20(1):53. doi: 10.3390/ijms20010053 (PMC6337286; doi:10.3390/ijms20010053)
Supplement: Supplementary file 1 [file ijms-20-00053-s001.pdf]

International Journal of
Molecular Sciences

MDPI

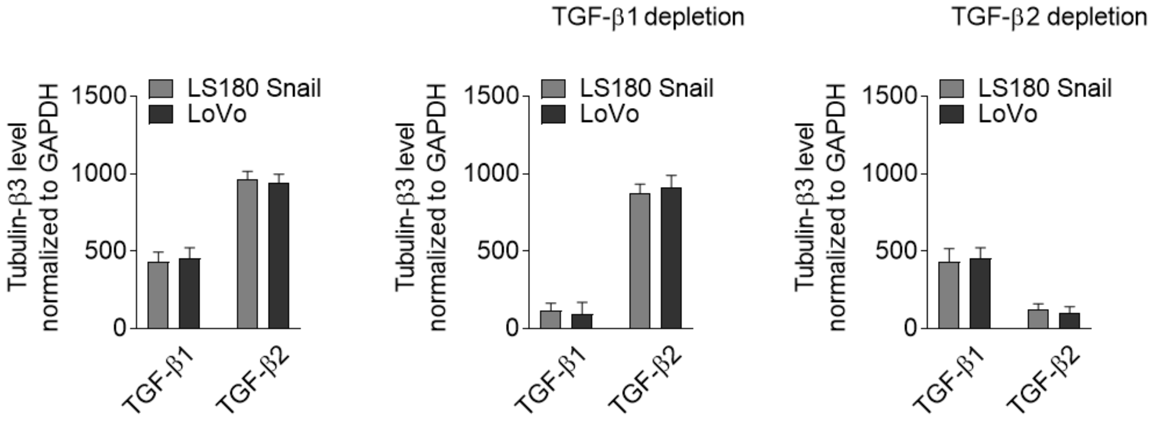

Figure S1. Conditioned medium from invasive colon cancer cells induces the early stages of EndMT in HMEC-1 cells. The level of TGF-β1 and TGF-β2 secreted into the medium was quantified by ELISA. The results are provided as mean ± SD (N=3). Methods of TGF-βs quantification in CM from colon cancer cell lines: The level of each TGF-β was measured by Quantikine ELISA Kits (R&D Systems, Minneapolis, MN) according to the manufacturer's procedure. The optical density of analyzed samples was measured at 450 nm using microplate reader (Bio-Rad, Hercules, CA) and corrected against-absorption at 570 nm.

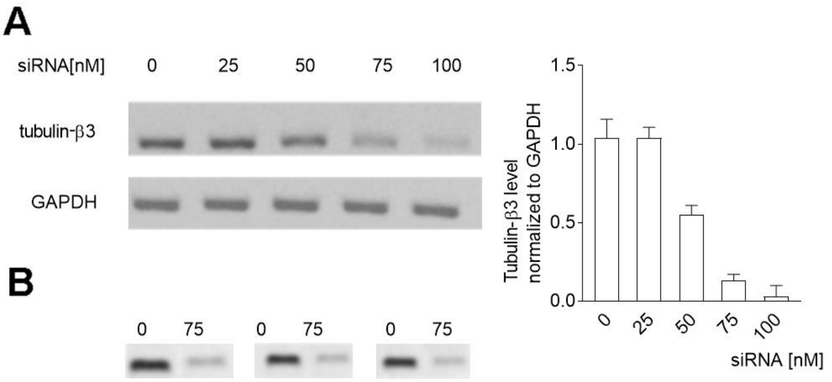

Figure S2. Effect of tubulin-β3 silencing in HMEC-1 stimulated cells. Analysis of siRNA anti-tubulin-β3 concentration silencing tested tubulin isoform by Western blot method. Level of tubulin-β3 in each sample was compared to GAPDH used as a loading control (A). Analysis of 75 nM siRNA treatment of the experimental probes in the three independent experiments (B). Level of tubulin-β3 in each sample was compared to GAPDH used as a loading control.

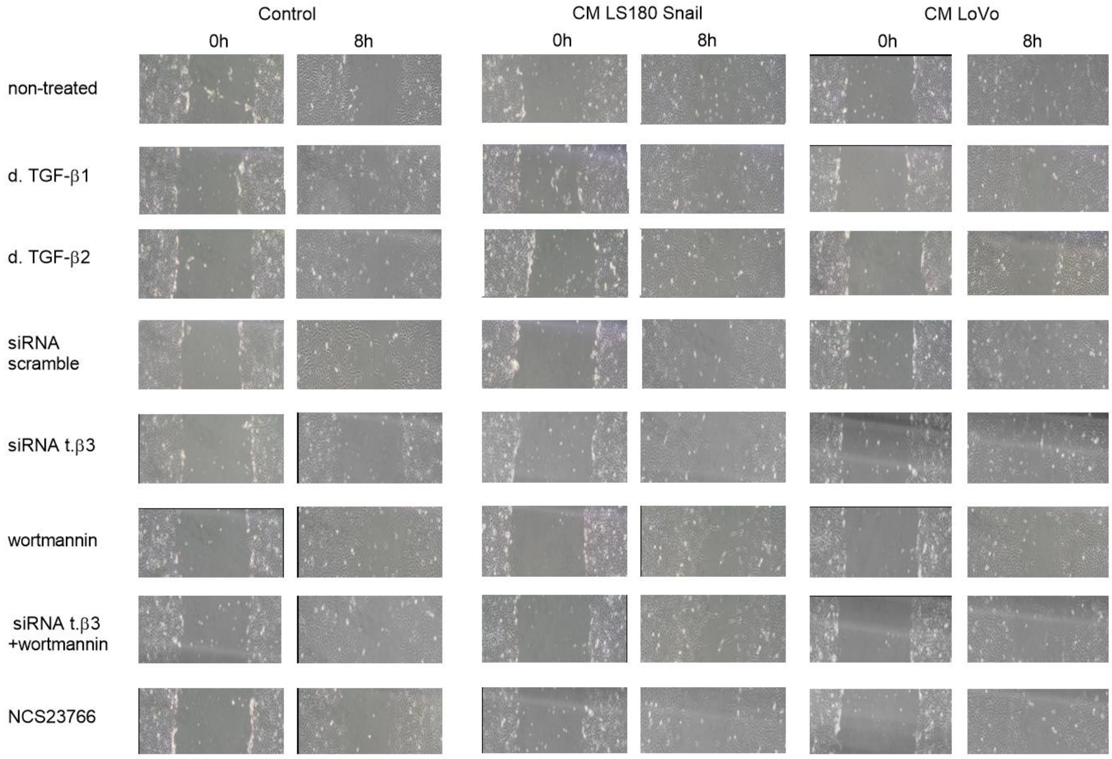

Figure S3. Phosphorylation of microtubular tubulin-β3 regulates CAFs behavior. HMEC-1 cells were cultured in medium supplemented with conditioned medium (CM) isolated from invasive (LS180-Snail, LoVo) colon cancer cells where TGF-β1 or TGF-β2 were depleted (d. TGF-β1 or d. TGF-β2) for 216 h or where tubulin-β3 was silenced (siRNA t. β3), phosphorylation of tubulin-β3 was inhibited by wortmannin. Additionally, in tubulin-β3-silenced cells (siRNA t. β3) the phosphorylation of tubulin-β3 was inhibited by wortmannin or Rac1 activity was inhibited by NCS23766. Then the wound healing properties were analyzed. Representative image were shown in the Figure above.

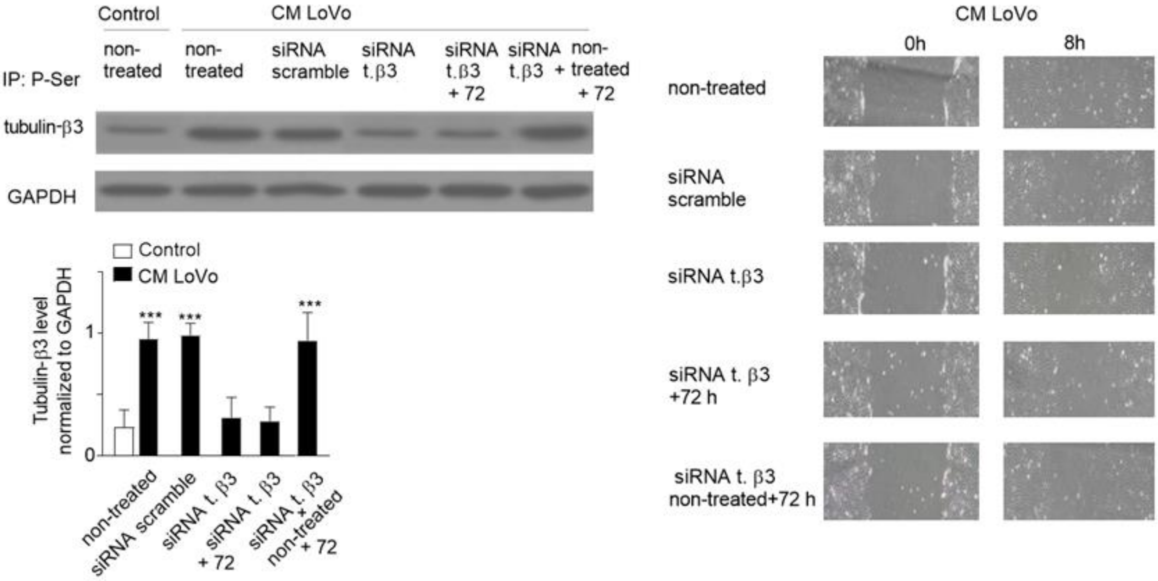

Figure S4. Expression of tubulin-β3 during recovery experiment. Level of tubulin-β3 expression in each experimental condition was analyzed by Western blot assay. GAPDH was used as a loading control. The results of three independent experiments are provided as mean ± SD (n = 3); *** $p < 0.005$. Then analysis of wound healing abilities was performed. The representative images of that assay were presented in the Figure.
